# Social Pressure-Induced Craving in Patients with Alcohol Dependence: Application of Virtual Reality to Coping Skill Training

**DOI:** 10.4306/pi.2008.5.4.239

**Published:** 2008-12-31

**Authors:** Jung Suk Lee, Kee Namkoong, Jeonghun Ku, Sangwoo Cho, Ji Yeon Park, You Kyong Choi, Jae-Jin Kim, In Young Kim, Sun I. Kim, Young-Chul Jung

**Affiliations:** 1 Department of Psychiatry and Institute of Behavioral Science in Medicine, Yonsei University College of Medicine, Seoul, Korea.; 2 Department of Biomedical Engineering, Hanyang University, Seoul, Korea.

**Keywords:** Alcohol cue, Social pressure, Virtual Reality

## Abstract

**Objective:**

This study was conducted to assess the interaction between alcohol cues and social pressure in the induction of alcohol craving.

**Methods:**

Fourteen male patients with alcohol dependence and 14 age-matched social drinkers completed a virtual reality coping skill training program composed of four blocks according to the presence of alcohol cues (×2) and social pressure (×2). Before and after each block, the craving levels were measured using a visual analogue scale.

**Results:**

Patients with alcohol dependence reported extremely high levels of craving immediately upon exposure to a virtual environment with alcohol cues, regardless of social pressure. In contrast, the craving levels of social drinkers were influenced by social pressure from virtual avatars.

**Conclusion:**

Our findings imply that an alcohol cue-laden environment should interfere with the ability to use coping skills against social pressure in real-life situations.

## Introduction

Social pressure, including both verbal and nonverbal persuasion, is known to create a high-risk situation that leads to relapse in alcohol dependence.^1^ Cognitive-behavioral coping skill training (e.g., refusing drink offers and assertive communication skills) has been suggested to offer the best protection against social pressure-induced relapse.^2^ Therefore, improvement of strategies for coping with drink offers is critical in reducing the risk of relapse in patients with alcohol dependence.^3^

While various efforts have been made to improve the efficacy of coping skill training, Monti et al.^4^ observed that drinkers who were exposed to alcohol cues performed poorly in drink refusal role-play situations and insisted that alcohol cues can disrupt an individual's ability to use coping skills. According to the social learning theory, alcohol cues can trigger cognitive and neurochemical reactions that may disrupt the individual's ability to use coping skills as well as the belief that one can effectively use those skills in the situation.^5^ There is now general agreement that coping skill training should be practiced in the presence of alcohol cues in order to increase both the effecttiveness and sense of self-efficacy in using coping skills when confronted with a similar real-life situation.^6^,^7^ However, there is great individual variation in the contexts associated with drinking, and social pressure is a difficult component to construct in treatment settings.

We developed a virtual reality (VR) system in order to assess the interaction between alcohol cues and social pressure in the induction of alcohol craving.^8^ We took advantage of virtual reality techniques that provide immersion environments and enable social interactions with virtual avatars.^9^,^10^ In our previous study,^8^ our VR system proved to induce craving in social drinkers through social pressure from virtual avatars. However, the presentation of an alcohol cue did not affect the social pressure-induced craving. In the present study, we applied our VR system to patients with alcohol dependence and hypothesized that patients with alcohol dependence would demonstrate different patterns of social pressure-induced craving according to the presence of alcohol cues.

## Methods

### Subjects

Fourteen male abstinent alcoholics and 14 age-matched healthy volunteers participated in this study (Table 1). In-patients with alcohol dependence who were enrolled in treatment programs and had abstained from alcohol consumption for at least three weeks were recruited for the alcoholic group. Participants were excluded if they had a lifetime history of comorbid psychiatric disorder, with the exception of nicotine dependence. Participants who drank fewer than 14 standard drinks, defined as any drink containing approximately 12 grams of ethanol, per week and fewer than four standard drinks per occasion were included in the control group, as long as they did not meet the criteria for lifetime alcohol use disorder. Two psychiatrists (L.S. and Y.J.) performed clinical interviews using the Structured Clinical Interview for the Diagnostic and Statistical Manual of Mental Disorder.^11^ Written informed consent was obtained from all subjects after they were given a complete description of the study. Our study was carried out under the guidelines for the use of human subjects established by the Institutional Review Board at Severance Mental Health Hospital.

### Virtual reality experiment and procedure

**FIGURE 1:**
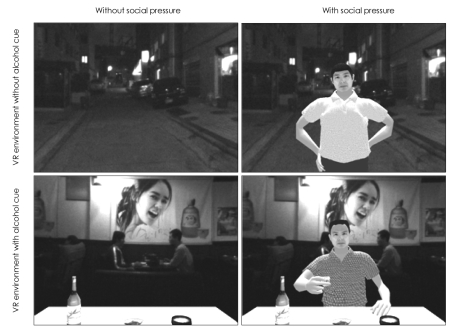
Virtual reality experiment. The VR experiment was composed of four blocks according to the presence of alcohol cues (×2) and social pressure (×2). During the blocks without social pressure, the participants were only exposed to the 3-dimensional environment. During the blocks with social pressure, a virtual avatar appeared and kept offering the participant a drink with persuasive arguments, such as "I broke up with my girlfriend, let's have a drink".

The development of the VR system was described in our previous study.^8^ Two VR environments were used in our VR task: one was a virtual street in which no alcohol cues were present, and the other was a virtual pub in which numerous alcohol cues were present (e.g., a glass filled with alcohol, a bottle, an advertisement for an alcoholic beverage). The VR experiment was composed of four blocks according to the presence of alcohol cues (×2) and social pressure (×2) as follows: 1) VR environment with no alcohol cues and no social pressure; 2) VR environment without alcohol cues but with social pressure; 3) VR environment with alcohol cues but without social pressure; and 4) VR environment with both alcohol cues and social pressure (Figure 1). Upon arriving the lab, participants were seated in a comfortable chair and asked to wear a head-mounted display (HMD) and tracker. After adjusting the HMD for comfort, participants were given a mouse to allow them to rate the level of alcohol craving. During the VR experiment, participants could see the computer-simulated three-dimensional environment and hear sounds from the speaker. Participants were first exposed to a VR environment without social pressure so that they would have the opportunity to look around and become familiarized with the 3-dimensional VR environment. After a 5-minute relaxation period, the participants were exposed to the same VR environment, but this time a virtual avatar appeared and kept offering the participant a drink with persuasive arguments, such as "I broke up with my girlfriend, let's have a drink." The craving levels were measured before and after each block on a visual analogue scale from "I do not want to drink (0%)" to "My desire to drink is unbearable (100%)". The block induced-craving were determined by calculating the difference between the craving rating before and after each VR block. The VR environments (virtual street, virtual pub) were assigned in a random order.

### Statistical analysis

We compared the alcohol craving levels measured in the presence and absence of social pressure through paired t-tests in each VR environment. We also performed repeated measures analysis of variance in order to examine the interaction effects: the craving level was dependent on the measure, with diagnosis (×2) defined as a between-group factor and the presence of alcohol cues (×2) or social pressure (×2) defined as a within-group factor. Statistical analyses were performed with the SPSS 15.0 package.

## Results

**FIGURE 2:**
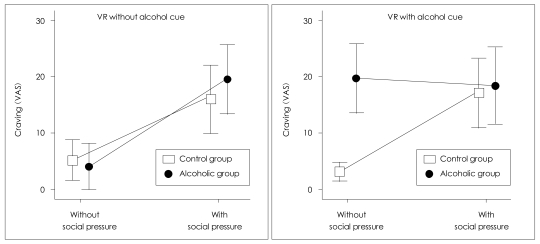
Induced craving in virtual reality environments with and without alcohol cues.

In the VR environment without alcohol cues (virtual street), alcohol cravings were significantly induced by social pressure in both groups (control group: t=-3.565, df=13, p=0.003; alcoholic group: t=-2.664, df=13, p=0.019). In the VR environment with alcohol cues (virtual pub), alcohol craving were significantly induced by social pressure in the control group (t=-2.602, df=13, p=0.022). Meanwhile, the alcoholic group demonstrated a distinct pattern of craving. The craving was strongly induced upon exposure to the virtual pub, even before social pressure was introduced. Interestingly, social pressure did not lead to further increases in the level of alcohol craving in the alcoholic group (t=0.339, df=13, p=0.740) (Figure 2).

There was a significant group×alcohol cue×social pressure interaction effect for craving level (F=8.143, df=1, p=0.008). The difference in the induction of craving in the VR environment with alcohol cues should have contributed to the significant interaction (Figure 2).

## Discussion

This study is the first to use the VR technique to assess the interaction between alcohol cues and social pressure in alcohol craving. We observed that the presence of alcohol cues induced an extremely high level of craving in the alcoholic group, which might lead to relapse in situations with social pressure.

Our findings have implications for combining cue-exposure and coping skill training in the treatment of alcohol dependence. Treatment approaches that combine coping skill training and cue exposure therapy have received growing attention.^6^ However, the high-risk situations associated with drinking vary among patients, and it is quite difficult to provide various contexts in which to practice coping skills in a treatment setting. More critically, learning during cue exposure therapy does not generalize across contexts if training takes place in only one context.^12^,^13^ Consistent with the findings of previous studies,^9^,^14^ our study findings suggest that the methodological usefulness of virtual reality, which might play significant roles in increasing the efficacy of cognitive behavioral treatments by presenting various emotional and social contexts that mimic real-life situations.

A difference between previous VR studies and the present study is the block design of the present study. Lee et al.^15^ used only alcohol cues to induce alcohol craving. The VR system developed by Bordnick et al.^9^ is composed of a block that contains social interaction situations, but there was no control block with which to compare the results. Thus, Bordnick et al. could not analyze the effect of social pressure on alcohol craving. In contrast, a social pressure situation was used to induce alcohol craving in this study, and our two-by-two experimental model was focused on analyzing the interaction between social pressure and alcohol cues and their effects on alcohol craving.

We hypothesized that the pattern of social pressure-induced craving would differ between alcoholic patients and social drinkers according to the presence of alcohol cues. Although the level of craving was significantly increased in the control group by social pressure from virtual avatars, it is noteworthy that, in the alcoholic group, social pressure did not lead to additional increases in the level of craving induced by exposure to a cue-laden environment. There are several possible explanations for these findings. First, ceiling effects are known to be especially relevant to craving assessment.^16^ Substantial evidence demonstrates that exposure to alcohol cues increases the level of alcohol craving in abstinent alcoholics.^17^,^18^ Alcoholic patients who rated their craving level near the endpoint of the scale when first exposed to the cue-laden VR environment could have failed to report the actual increase in the level of craving caused by social pressure. In addition, we cannot rule out the possibility that the strong cognitive and neurochemical reactions to alcohol cues should have interfered with the ability to accurately assess one's internal state.^5^ Because there was no difference in the level of craving between the two groups in the VR environment without alcohol cues, our findings indicate that patients with alcohol dependence are vulnerable to alcohol-cue laden environments. Limitations of the visual analogue scale should be considered. Fox et al.^19^ reported that alcohol cue-induced craving and stress-induced craving were associated with dissociable psychobiological states. It is plausible to assume that social pressure-induced craving and cue-induced craving are related to different components of craving, and therefore a single-item scale might have been insufficient to assess the synergistic interaction between alcohol cue and social pressure. Various measures (self-report, physiological, and behavioral) are required in order to track the different components of cue-reactivity.^20^

In conclusion, our findings imply that the presence of alcohol cue-induced craving, regardless of social pressure, should interfere with the ability to use coping skill against social pressure in real-life situations. The role of virtual reality techniques in coping skill training should be expanded by providing various emotional and social contexts that mimic real-life situations.

## Figures and Tables

**TABLE 1:**
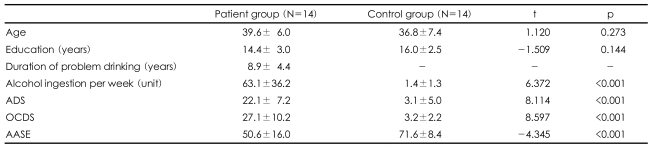
Demographic data and clinical profiles

ADS: Alcohol Dependence Scale^21^, OCDS: Obsessive-Compulsive Drinking Scale^22^, AASE: Alcohol Abstinence Self Efficacy Scale^23^
